# Supplementary material for: Quantum tunneling during interstellar surface-catalyzed formation of water: the reaction H + H2O2 → H2O + OH
Source: Phys Chem Chem Phys. 2016 Nov 8;18(48):33021–30. doi: 10.1039/c6cp06457d (PMC5317215; doi:10.1039/c6cp06457d)
Supplement: Supplementary file 1 [file CP-018-C6CP06457D-s001.pdf]

Electronic Supplementary Material (ESI) for Physical Chemistry Chemical Physics.
This journal is © the Owner Societies 2016

Electronic Supplementary Material accompanying Quantum tunneling during interstellar surface-catalyzed formation of water: the reaction $H + H_2O_2 \longrightarrow H_2O + OH$

Thanja Lamberts[1], Pradipta Kumar Samanta, Andreas Köhn, and Johannes Kästner
Institute of Theoretical Chemistry, University of Stuttgart, Stuttgart, Germany. Tel: +49 (0)711 685 64833

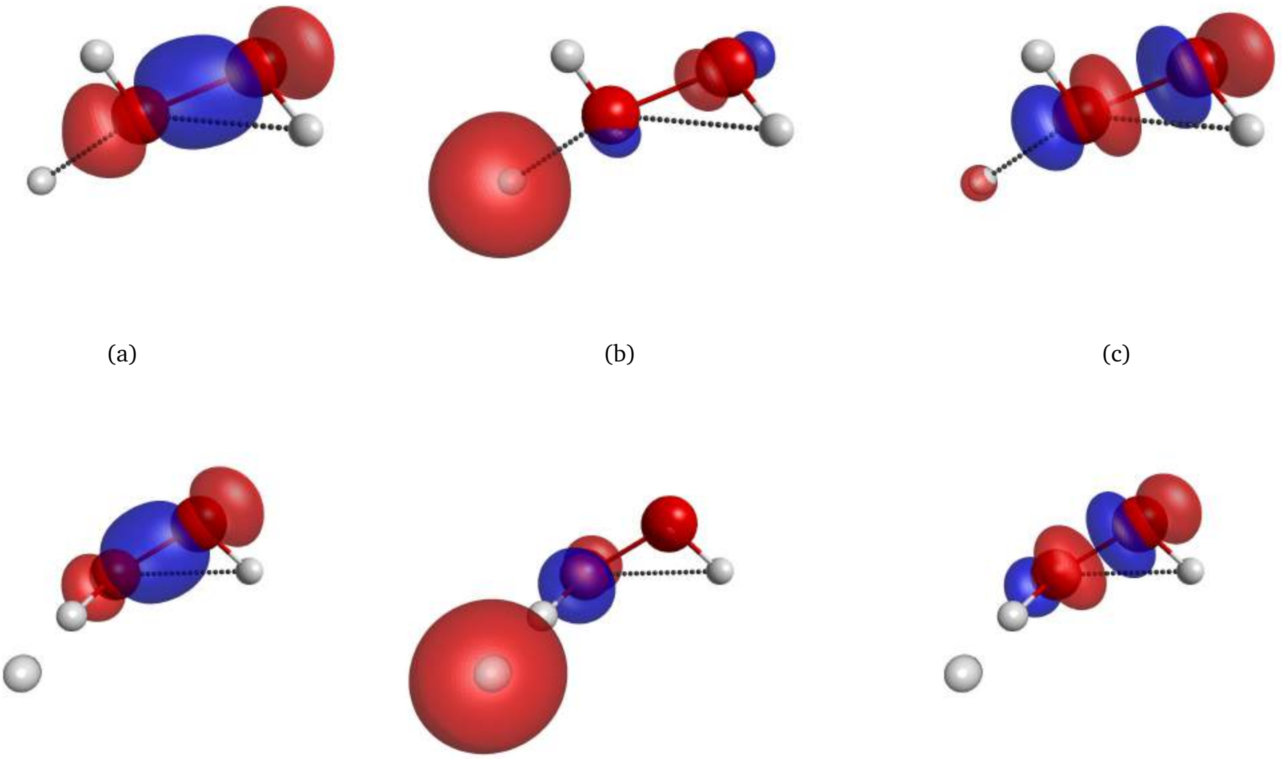

(a) (b) (c) (d) (e) (f)

Figure 1 Visualization of the orbitals that contribute to the main configurations to the CASSCF wavefunction for the transition states. Panels (a)-(c) concern the reaction $H + H_2O_2 \longrightarrow H_2O + OH$, (d)-(f) concern $H + H_2O_2 \longrightarrow HO_2 + H_2$. More specifically, panels (a) and (d) refer to the doubly occupied bonding sigma orbital, (b) and (e) to the singly occupied orbital with the main contribution from the hydrogen 1s, and (c) and (f) correspond to the doubly occupied anti-bonding sigma orbital.

[1]Corresponding author, E-mail: lamberts@theochem.uni-stuttgart.de

Table 1 Coordinates of stationary points in the gas-phase reactions in Å.

R1: $H + H_2O_2 \longrightarrow H_2O + OH$, MPW1B95/MGS											
H_2O_2				PRC				TS1			
O	-2.566047	0.007215	-1.672615	O	-2.523978	0.059220	-1.915333	O	-2.456521	0.538181	-0.592586
O	-2.560618	0.459818	-0.325739	O	-2.537889	0.491148	-0.561685	O	-2.649201	-0.051250	-1.972562
H	-1.774615	0.027550	0.016493	H	-3.312566	-0.487467	-1.948096	H	-3.509128	-0.462642	-1.845152
H	-3.352004	-0.544067	-1.684347	H	-1.759731	0.049346	-0.213814	H	-1.668072	0.055232	-0.332188
				H	-3.314133	-1.480655	-4.901822	H	-2.516195	-0.515257	-3.485223
H_2O				OH							
O	-0.984226	0.010059	0.010222	O	-4.417126	-1.035022	-3.648612				
H	-0.984226	0.956868	-0.116238	H	-4.417126	-0.118977	-3.333217				
H	-0.984226	-0.116510	0.957017								

R2 $H + H_2O_2 \longrightarrow H_2 + HO_2$, M05-2X/MGS											
H_2O_2								TS2			
O	-2.566047	0.007215	-1.672615					O	-2.540125	-0.086097	-1.981867
O	-2.560618	0.459818	-0.325739					O	-2.428133	0.423187	-0.702057
H	-1.774615	0.027550	0.016493					H	-3.582191	-0.471690	-2.053260
H	-3.352004	-0.544067	-1.684347					H	-1.992564	-0.286630	-0.218672
								H	-4.505798	-0.872040	-2.069527
HO_2				H_2							
O	-2.564350	0.023913	-1.617960	H	-4.417126	-0.948657	-3.648560				
O	-2.561233	0.438269	-0.379893	H	-4.417126	-0.207583	-3.648560				
H	-1.757099	0.073540	0.020414								

Table 2 Coordinates of stationary points of the reaction $H + H_2O_2 \longrightarrow H_2O + OH$ with spectator molecules in Å. The labeling refers to Fig. 4 of the paper.

One spectator water molecule

$H_2O_2-H_2O$ A			
O	-5.125237	-0.357020	-0.778591
H	-4.585890	0.177561	-0.190993
H	-5.506749	-1.044859	-0.236535
O	-2.576310	-0.173817	-1.894599
O	-2.419354	0.529414	-0.666455
H	-3.490102	-0.482836	-1.801743
H	-1.697906	0.043091	-0.261665

$PRC-H_2O$ A			
O	-5.123255	-0.499367	-0.864485
H	-4.687525	0.048184	-0.206667
H	-5.540549	-1.213000	-0.385296
O	-2.510508	-0.011082	-1.719634
O	-2.547431	0.602982	-0.434853
H	-3.386325	-0.425991	-1.741413
H	-1.853862	0.124421	0.025295
H	-5.620533	-1.185713	-5.782503

$TS-H_2O$ A			
O	-5.048236	-0.386637	-0.764551
H	-4.482890	0.077572	-0.142012
H	-5.398800	-1.141630	-0.295854
O	-2.594953	-0.059260	-1.996704
O	-2.394081	0.581132	-0.644841
H	-3.505927	-0.377126	-1.866834
H	-1.631230	0.078254	-0.350556
H	-2.472927	-0.546619	-3.522098

$H_2O_2-H_2O$ B			
O	-0.318076	-1.212914	-0.570619
H	0.572205	-0.922624	-0.761098
H	-0.756050	-1.294641	-1.421550
O	-2.632015	-0.187597	-1.869851
O	-2.475941	0.557472	-0.667333
H	-3.480492	-0.611818	-1.722743
H	-1.707185	0.109292	-0.282768

$TS-H_2O$ B			
O	-0.366035	-1.207674	-0.632668
H	0.549438	-0.972658	-0.773722
H	-0.747114	-1.322406	-1.505075
O	-2.688664	-0.103423	-1.955435
O	-2.492581	0.635180	-0.642629
H	-3.554133	-0.488517	-1.788891
H	-1.713126	0.145986	-0.334401
H	-2.638670	-0.530672	-3.454237

Two spectator water molecules

$H_2O_2-H_2O$ A and B			
O	-5.052208	-0.411026	-0.762361
H	-4.471248	0.098995	-0.189741
H	-5.373107	-1.141146	-0.236406
O	-0.374193	-1.210476	-0.616959
H	0.530795	-0.990987	-0.830916
H	-0.831642	-1.294604	-1.457202
O	-2.570985	-0.089453	-1.970360
O	-2.442095	0.640165	-0.752382
H	-3.467036	-0.445645	-1.867521
H	-1.687747	0.180127	-0.353156

$PRC-H_2O$ A and B			
O	-5.052208	-0.411026	-0.762361
H	-4.471248	0.098995	-0.189741
H	-5.373107	-1.141146	-0.236406
O	-0.374193	-1.210476	-0.616959
H	0.530795	-0.990987	-0.830916
H	-0.831642	-1.294604	-1.457202
O	-2.573700	-0.104418	-1.970376
O	-2.443565	0.633823	-0.757516
H	-3.472348	-0.454193	-1.865314
H	-1.686892	0.177263	-0.358348
H	-3.530839	-1.479906	-4.928228

$TS-H_2O$ A and B			
O	-5.052208	-0.411026	-0.762361
H	-4.471248	0.098995	-0.189741
H	-5.373107	-1.141146	-0.236406
O	-0.374193	-1.210476	-0.616959
H	0.530795	-0.990987	-0.830916
H	-0.831642	-1.294604	-1.457202
O	-2.614762	-0.105739	-1.983349
O	-2.467158	0.651642	-0.678011
H	-3.519935	-0.441149	-1.848034
H	-1.691103	0.179774	-0.336076
H	-2.535703	-0.515154	-3.508159

Three spectator water molecules

$H_2O_2-H_2O$ A B and C			
O	-4.806379	-0.676185	-1.020481
H	-5.442425	-0.105454	-0.592480
H	-4.359460	-1.154685	-0.309710
O	-0.757202	-1.608931	-0.896299
H	0.145162	-1.376953	-0.685917
H	-1.153507	-0.819453	-1.291686
O	-2.851068	-1.609262	0.830244
H	-2.841822	-2.123021	1.635008
H	-2.047368	-1.837413	0.328419
O	-2.394200	0.566795	-1.381494
O	-2.465767	1.123051	-0.076254
H	-3.308474	0.254573	-1.515981
H	-2.501589	0.334944	0.484601

$PRC-H_2O$ A B and C			
O	-4.812028	-0.721008	-1.033874
H	-5.545431	-0.247308	-0.646236
H	-4.361410	-1.165212	-0.304065
O	-0.807758	-1.610371	-0.947913
H	0.119664	-1.432568	-0.806421
H	-1.200998	-0.784148	-1.274892
O	-2.835769	-1.548592	0.849564
H	-2.812565	-2.064158	1.652907
H	-2.049155	-1.790900	0.325681
O	-2.416444	0.602948	-1.352822
O	-2.480406	1.190758	-0.061981
H	-3.331310	0.292281	-1.482807
H	-2.512060	0.414811	0.515376
H	-3.301150	-0.958459	-4.559923

$TS-H_2O$ A B and C			
O	-4.806379	-0.676185	-1.020481
H	-5.442425	-0.105454	-0.592480
H	-4.359460	-1.154685	-0.309710
O	-0.757202	-1.608931	-0.896299
H	0.145162	-1.376953	-0.685917
H	-1.153507	-0.819453	-1.291686
O	-2.851068	-1.609262	0.830244
H	-2.841822	-2.123021	1.635008
H	-2.047368	-1.837413	0.328419
O	-2.445660	0.615431	-1.450338
O	-2.501926	1.178943	-0.037112
H	-3.357284	0.256582	-1.515001
H	-2.486160	0.355436	0.471795
H	-2.115470	0.398469	-2.958102

Table 3 Gas-phase bimolecular rate constants (cm^3 s^{-1}) for hydrogenation and deuteration of peroxide.

Temp. (K)	$H + H_2O_2 \longrightarrow H_2O + OH$	$H + D_2O_2 \longrightarrow HDO + OD$	$D + H_2O_2 \longrightarrow HDO + OH$	$D + D_2O_2 \longrightarrow D_2O + OD$	$H + H_2^{18}O_2 \longrightarrow H_2^{18}O + {}^{18}O$	$H + H_2O_2 \longrightarrow H_2 + HO_2$
50.3	3.69E-19	2.74E-19	2.07E-21	1.57E-21	2.58E-19	
54.7	3.68E-19	2.85E-19	2.36E-21	1.78E-21	2.64E-19	
59.9	4.64E-19	3.75E-19	3.49E-21	2.63E-21	3.48E-19	
66.2	6.70E-19	5.44E-19	5.04E-21	3.85E-21	4.81E-19	
74.0	7.70E-19	6.11E-19	8.33E-21	6.45E-21	5.71E-19	
83.8	1.21E-18	9.66E-19	1.68E-20	1.29E-20	9.21E-19	
96.7	1.83E-18	1.47E-18	3.97E-20	3.10E-20	1.36E-18	
114.2	3.78E-18	3.09E-18	1.30E-19	1.03E-19	2.88E-18	2.31E-20
139.4	9.87E-18	8.13E-18	7.06E-19	5.61E-19	7.64E-18	1.39E-19
179.0	4.81E-17	4.01E-17	9.85E-18	8.00E-18	3.85E-17	2.12E-18
200.0	9.60E-16	8.15E-16			8.63E-16	
250.0			3.95E-17	3.27E-17		1.08E-16
414.3						1.80E-14
450.0						3.84E-14

Table 4 Eley-Rideal bimolecular rate constants (cm^3 s^{-1}) for hydrogenation and deuteration of peroxide with and without spectator H_2O molecules.

Temp. (K)	No H_2O	1 H_2O A	1 H_2O B	2 H_2O	3 H_2O
			$H + H_2O_2 \longrightarrow H_2O + OH$		
50.3	1.09E-19	3.14E-19	3.81E-20	8.63E-20	1.85E-20
54.7	1.09E-19	3.00E-19	4.74E-20	1.11E-19	1.53E-20
59.9	1.39E-19	3.16E-19	5.54E-20	1.28E-19	1.80E-20
66.2	2.02E-19	7.99E-19	6.11E-20	1.69E-19	2.32E-20
74.0	2.35E-19	6.39E-19	7.93E-20	2.55E-19	2.85E-20
83.8	3.74E-19	1.01E-18	1.23E-19	3.45E-19	3.86E-20
96.7	5.72E-19	1.68E-18	2.07E-19	5.26E-19	5.71E-20
114.2	1.20E-18	3.72E-18	4.03E-19	9.19E-19	1.14E-19
139.4	3.18E-18	1.09E-17	1.11E-18	2.81E-18	3.10E-19
179.0	1.58E-17	5.37E-17	5.90E-18	1.51E-17	1.66E-18
250.0	3.18E-16	9.87E-16	1.36E-16	2.72E-16	4.37E-17
			$D + H_2O_2 \longrightarrow HDO + OH$		
50.3	4.76E-22			4.28E-22	3.55e-23
54.7	5.49E-22			5.50E-22	4.99e-23
59.9	8.21E-22			7.30E-22	6.69e-23
66.2	1.20E-21			1.14E-21	8.72E-23
74.0	2.01E-21			1.99E-21	1.39E-22
83.8	4.11E-21			3.98E-21	2.64E-22
96.7	9.92E-21			9.98E-21	6.50E-22
114.2	3.31E-20			3.47E-20	2.27E-21
139.4	1.84E-19			2.09E-19	1.36E-20
179.0	2.62E-18			3.10E-18	2.31E-19
200.0	1.06E-17			1.28E-17	1.10E-18

Table 5 Langmuir-Hinshelwood unimolecular rate constants (s^{-1}) for hydrogenation and deuteration of peroxide with and without spectator H_2O molecules.

Temp. (K)	No H_2O	1 H_2O A	2 H_2O	3 H_2O
		$H + H_2O_2 \longrightarrow H_2O + OH$		
50.3	3.18E+04	5.71E+04		8.32E+03
54.7	2.66E+04	4.70E+04	3.17E+04	
59.9	2.82E+04	4.24E+04		5.84E+03
66.2	3.39E+04	9.07E+04	2.42E+04	6.36E+03
74.0	3.22E+04	6.06E+04	2.61E+04	6.56E+03
83.8	4.13E+04	7.86E+04	2.76E+04	7.37E+03
96.7	5.02E+04	1.05E+05	3.74E+04	8.95E+03
114.2	8.14E+04	1.81E+05	5.43E+04	1.43E+04
139.4	1.60E+05	3.96E+05	1.18E+05	3.01E+04
179.0	5.52E+05	1.36E+06	3.99E+05	1.18E+05
250.0	6.92E+06	1.54E+07	4.99E+06	2.02E+06
		$D + H_2O_2 \longrightarrow HDO + OH$		
50.3	1.61E+02		8.53E+01	6.96e+00
54.7	1.63E+02		9.33E+01	9.18e+00
59.9	2.11E+02		1.05E+02	1.14e+01
66.2	2.67E+02		1.38E+02	1.38E+01
74.0	3.80E+02		2.00E+02	2.01E+01
83.8	6.53E+02		3.28E+02	3.43E+01
96.7	1.29E+03		6.63E+02	7.46E+01
114.2	3.44E+03		1.81E+03	2.23E+02
139.4	1.45E+04		8.20E+03	1.10E+03
179.0	1.47E+05		8.54E+04	1.43E+04
200.0	5.12E+05		3.03E+05	6.00E+04
